# Effectiveness of Virtual Simulations Versus Mannequins and Real Persons in Medical and Nursing Education: Meta-Analysis and Trial Sequential Analysis of Randomized Controlled Trials

**DOI:** 10.2196/56195

**Published:** 2024-12-05

**Authors:** Nan Jiang, Yuelun Zhang, Siyu Liang, Xiaohong Lyu, Shi Chen, Xiaoming Huang, Hui Pan

**Affiliations:** 1 4 + 4 Medical Doctor Program Peking Union Medical College Hospital Chinese Academy of Medical Sciences and Peking Union Medical College Beijing China; 2 Medical Research Center Peking Union Medical College Hospital Chinese Academy of Medical Sciences and Peking Union Medical College Beijing China; 3 Department of Endocrinology Key Laboratory of Endocrinology of National Health Commission Translation Medicine Centre, Peking Union Medical College Hospital, Peking Union Medical College, Chinese Academy of Medical Sciences Beijing China; 4 Department of Breast Surgery Peking Union Medical College Hospital Chinese Academy of Medical Sciences and Peking Union Medical College Beijing China; 5 Department of Family Medicine & Division of General Internal Medicine Peking Union Medical College Hospital Chinese Academy of Medical Sciences and Peking Union Medical College Beijing China

**Keywords:** artificial intelligence, clinical virtual simulation, medical education, meta-analysis, nursing education, virtual patient, virtual reality

## Abstract

**Background:**

Virtual simulation (VS) is a developing education approach with the recreation of reality using digital technology. The teaching effectiveness of VSs compared to mannequins and real persons (RPs) has never been investigated in medical and nursing education.

**Objective:**

This study aims to compare VSs and mannequins or RPs in improving the following clinical competencies: knowledge, procedural skills, clinical reasoning, and communication skills.

**Methods:**

Following Cochrane methodology, a meta-analysis was conducted on the effectiveness of VSs in pre- and postregistration medical or nursing participants. The Cochrane Library, PubMed, Embase, and Educational Resource Information Centre databases were searched to identify English-written randomized controlled trials up to August 2024. Two authors independently selected studies, extracted data, and assessed the risk of bias. All pooled estimates were based on random-effects models and assessed by trial sequential analyses. Leave-one-out, subgroup, and univariate meta-regression analyses were performed to explore sources of heterogeneity.

**Results:**

A total of 27 studies with 1480 participants were included. Overall, there were no significant differences between VSs and mannequins or RPs in improving knowledge (standard mean difference [SMD]=0.08; 95% CI –0.30 to 0.47; *I*^2^=67%; *P*=.002), procedural skills (SMD=–0.12; 95% CI –0.47 to 0.23; *I*^2^=75%; *P*<.001), clinical reasoning (SMD=0.29; 95% CI –0.26 to 0.85; *I*^2^=88%; *P*<.001), and communication skills (SMD=–0.02; 95% CI: –0.62 to 0.58; *I*^2^=86%; *P*<.001). Trial sequential analysis for clinical reasoning indicated an insufficient sample size for a definitive judgment. For procedural skills, subgroup analyses showed that VSs were less effective among nursing participants (SMD=–0.55; 95% CI –1.07 to –0.03; *I*^2^=69%; *P*=.04). Univariate meta-regression detected a positive effect of publication year (β=.09; *P*=.02) on communication skill scores.

**Conclusions:**

Given favorable cost-utility plus high flexibility regarding time and space, VSs are viable alternatives to traditional face-to-face learning modalities. The comparative effectiveness of VSs deserves to be followed up with the emergence of new technology. In addition, further investigation of VSs with different design features will provide novel insights to drive education reform.

**Trial Registration:**

PROSPERO CRD42023466622; https://www.crd.york.ac.uk/prospero/display_record.php?RecordID=466622

## Introduction

The ultimate goal of health profession education is to promote the transfer of theoretical knowledge into clinical practice. Studies in cognitive psychology suggest that recall and application of information are best in learning environments similar to workplace [1]. Clinical simulation, a technique that replicates real experiences with guided experiences, has thereby gained popularity in the last 2 decades and is currently the main tool for training health care professionals [2]. There are five main categories of simulation: (1) low-tech simulators (ie, models or mannequins); (2) standardized patients (SPs); (3) screen-based computer programs; (4) high-fidelity computer simulators integrated with visual, audio, and touch cues; and (5) computer-driven, full-length mannequins with realistic anatomy and physiology [3]. With the help of simulation, health care professionals refine the knowledge, skills, and attitudes needed to deliver quality patient care while patients are protected from unnecessary risk [3]. In medical and nursing education, simulation-based teaching has demonstrated superior effectiveness compared to didactic teaching [4,5]; moreover, it reduces anxiety and increases the confidence of students entering practice [6,7]. While simulation-based education may bring downstream benefits of reducing medical errors and improving patient care, the current level of evidence is still low due to a lack of high-quality studies with patient-centered outcomes [8-12].

Driven by COVID-19 social distancing and modern technological advancement, there has been a marked shift toward learning on digital platforms or software. Virtual simulation (VS) refers to the 2D or 3D recreation of reality by digital technology, where students are able to interact with digital scenes, instruments, and characters [13,14]. Compared to conventional simulations (eg, mannequins and SPs), VSs can be undertaken flexibly with no limit of time and space [15]; they can provide a more realistic experience with the aid of artificial intelligence (AI) and virtual reality (VR) when certain pathological findings cannot be readily expressed [16-18]. For educators, VSs can potentially save costs due to reduced instructor time, manpower, and space resources [19]. One recent analysis found that VR simulations required 22% less time and 40% lower cost than traditional high-fidelity simulations to achieve the same learning outcomes [20]. Documentation and evaluation of student performance can also be automated with digital technologies [19].

Despite these promising advantages, the comparative effectiveness of training health professionals using mannequins or real persons (RPs; eg, SPs, role-play, actual patients) and VSs remains uncertain. Previous meta-analyses have shown that compared to traditional education (eg, lectures, reading exercises, in-class group discussions, mannequins, SPs), digital education is at least as effective in training medical students’ communication skills [15]; virtual patients (VPs) can more effectively improve skills and can be at least as effective in enhancing knowledge [21]; VR can more effectively improve not only skills but also knowledge [22]. The comparison with an extensive category of “traditional education” is insufficient to determine the value and viability of implementing VS as substitutes for mannequins and RPs, so specific recommendations for educational reform cannot be made. Thus, this study aims to investigate the teaching efficacy of VSs versus mannequins and RPs in a fine-grain spectrum of clinical competencies, including knowledge, procedural skills, clinical reasoning, and communication skills.

## Methods

### Reporting Guidelines

This study follows the PRISMA (Preferred Reporting Items for Systematic Reviews and Meta-analyses) guidelines [23]. Multimedia Appendix 1 provides a PRISMA checklist of this meta-analysis. The protocol was registered in PROSPERO (CRD42023466622).

### Eligibility Criteria

This study included randomized controlled trials (RCTs) from inception to August 2024. Inclusion and exclusion criteria are listed in Textbox 1.

**Inclusion and exclusion criteria.**

Inclusion criteria

- Year: From inception to August 2024
- Study design: Randomized controlled trials
- Language: English
- Pre- and post-registration medical/nursing students and staff
- Intervention: Virtual simulations
- Comparison: Mannequins or real persons
- Outcome: Objective posttest ratings for knowledge, procedural skills, clinical reasoning, or communication skills
- Data availability: Yes

Exclusion criteria

- Intervention: Blended simulations where virtual simulations and mannequins or real persons were both applied
- Outcome: Self-evaluations

The four outcomes of interest were defined as follows: (1) knowledge: remembering and understanding basic concepts, measured by question-based theoretical tests [24]; (2) procedural skills: following a series of steps to accomplish certain tasks, demonstrated by simulation exams [24]; (3) clinical reasoning: evaluating and reacting to clinical situations, demonstrated by simulation examinations [24]; and (4) communication skills: Interacting with patients or colleagues verbally and nonverbally, demonstrated by simulation examinations [24].

### Search Strategy

A thorough search was carried out in the Cochrane Library, PubMed, Embase, and Educational Resource Information Centre databases by specifying the desired population, intervention, and comparison. Search terms were selected judiciously, with specific terms customized for each database (Multimedia Appendix 2). Additionally, we searched Google and reference lists of the selected studies to retrieve other relevant publications.

Search records were imported into the EndNote library (version X9; Clarivate). After eliminating duplicates, the remaining studies underwent eligibility screening by 2 independent reviewers (NJ and SYL) according to predefined inclusion and exclusion criteria (Textbox 1). The initial screening process involved the assessment of titles and abstracts for relevance. Subsequently, full-text screening was performed, and the rationale for exclusion was documented in the PRISMA flowchart. In cases of discrepancies between the 2 reviewers, a third reviewer (SC) was consulted to reach a consensus. Study authors were contacted for crucial missing information. Finally, all researchers agreed on the final literature to be included in the analysis.

### Data Extraction

Two reviewers (NJ and SYL) independently extracted data in a structured form in Microsoft Excel, including author, publication date, country, participants, sample size, type of intervention, type of comparison, and outcomes. If a study had multiple posttest measurements, only the first measurement was recorded due to concern about the learning effect. If participants were rated by both SPs and independent evaluators, the ratings of the latter were preferred. If an outcome was assessed by multiple rating scales, the scale with the highest intraclass correlation coefficient was chosen. If an outcome was reported as multiple items instead of a total score, the primary item was selected, and if that was impossible, the mean score of all items was calculated. If a study had both mannequin and RP as control groups, each pairwise comparison was included separately while splitting the shared intervention group approximately evenly among the comparisons. The outcomes were recorded as mean values and SDs. For studies that reported outcomes as median values and IQRs or as mean values and CIs, these measures were converted to mean values and SDs using methods described in previous literature [25,26]*.*

### Risk of Bias Assessment

Two reviewers (NJ and SYL) independently assessed the risk of bias of the included studies using the Cochrane tool [25]. The following domains were considered: random sequence generation, allocation sequence concealment, blinding of participants or personnel, blinding of outcome assessment, complete outcome data, selective reporting, and other sources of bias. Discrepancies were resolved by discussion with a third author. Studies were not excluded from data extraction or analysis based on bias assessment scores.

### Statistical Analysis

Since data were measured using different tools, the mean differences were recalculated into standardized mean differences (SMDs). The results are displayed in forest plots, with pooled SMDs computed using random-effects meta-analysis models. We interpreted the effect size as small (SMD=0.2), moderate (SMD=0.5), or large (SMD=0.8) [25]. Publication bias was assessed by the Egger regression asymmetry test.

Cochran *Q* (chi-square test) was used to evaluate statistical heterogeneity, with a statistical significance threshold at *P*<.10. *I*^2^ statistic was adopted to quantify the degree of heterogeneity, where *I*^2^ was categorized as unimportant (0%-40%), moderate (30%-60%), substantial (50%-90%), and considerable (75%-100%) [25]. To investigate possible sources of heterogeneity, the leave-one-out method was first applied. Next, for each outcome, we conducted subgroup analyses by discipline (medicine; nursing), level (undergraduate; nonundergraduate [graduate students or clinical staff]), and comparison (mannequin; RP) to see if *I*^2^<50% in both groups [27]. Univariate random-effects meta-regression analyses were then performed to investigate whether heterogeneity between trials could be attributed to year of publication, age of participants, discipline, level, and comparison. Multivariate meta-regression was not performed due to the limited number of studies and the risk of overfitting.

Trial sequential analysis (TSA) was used to evaluate the strength of evidence and adjusted for potential errors. The analysis set specific values for type 1 and 2 errors (5% and 20%) and used these values to calculate trial sequential monitoring boundaries, futility boundaries, and the required information size (RIS) [28]. The mean difference to generate RIS was set to detect a mean difference of 2.0 between intervention and comparison. The variance was estimated by pooling all included trials. Heterogeneity was adjusted based on the estimated ratio between the variance in the random-effects model and the variance in the fixed-effects model. Finally, a graphical evaluation was used to determine if the cumulative *Z* curve met defined thresholds.

All data analyses were performed using package meta of R software (version 4.2.3; R Foundation for Statistical Computing) [29] and TSA software (version 0.9.5.10 beta; Copenhagen Trial Unit). Two-sided *P* values<.05 were considered to be statistically significant.

## Results

### Included Studies

**Figure 1:**
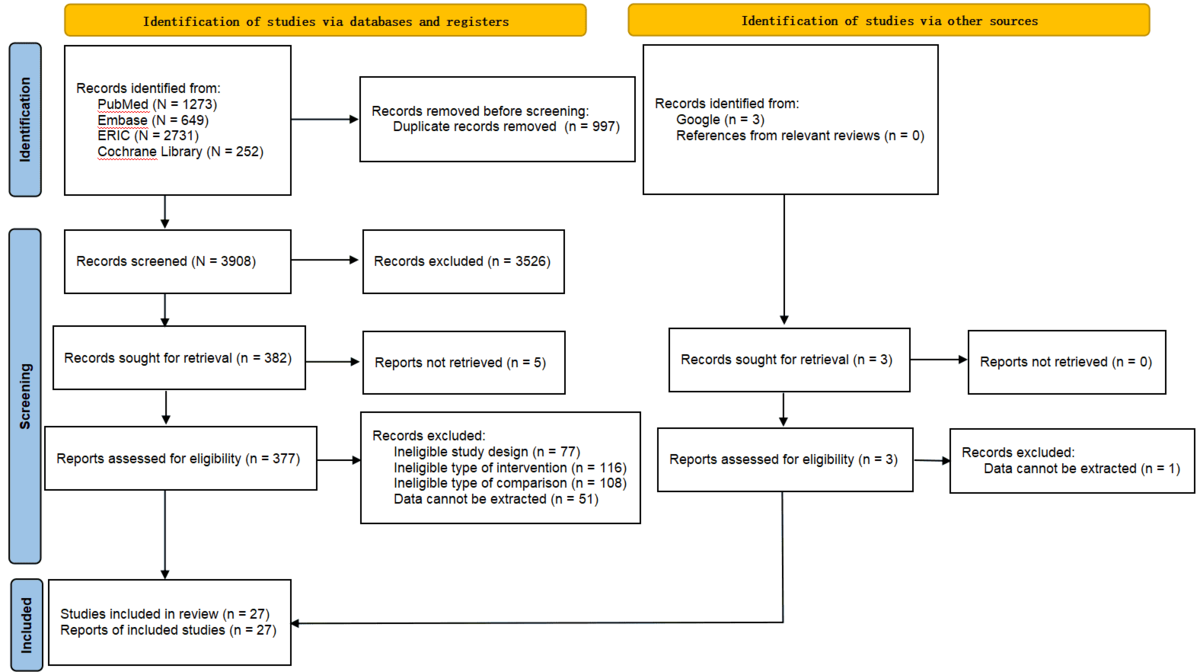
PRISMA (Preferred Reporting Items for Systematic Reviews and Meta-analyses) flow diagram.

After the selection process, a total of 27 studies with 1480 participants were included (Figure 1). In terms of discipline, participants in 14 studies were from the field of medicine [16,18,30-41]; participants in 11 studies were from the field of nursing [42-52]; and the remaining 2 studies enrolled mixed participants from medicine and nursing [53,54]. In terms of level, participants in 14 studies were undergraduate students [16,31,35,39,42-46,50-54]; participants in 8 studies were graduate students or clinical staff [18,32-34,36-38,40,41,49]; and the remaining 2 studies enrolled mixed undergraduate and nonundergraduate participants [30,48]. In terms of comparison, 13 studies compared VSs and mannequins [33-36,38,40,44-51]; 10 studies compared VSs and RPs [16,18,30,31,37,39,41-43,52-54]; and the remaining study included mannequins and RPs as comparison groups simultaneously [32]. Detailed information and extracted data for each study are summarized in Multimedia Appendix 3 [16,18,30-54]*.*

### Risk of Bias

**Figure 2:**
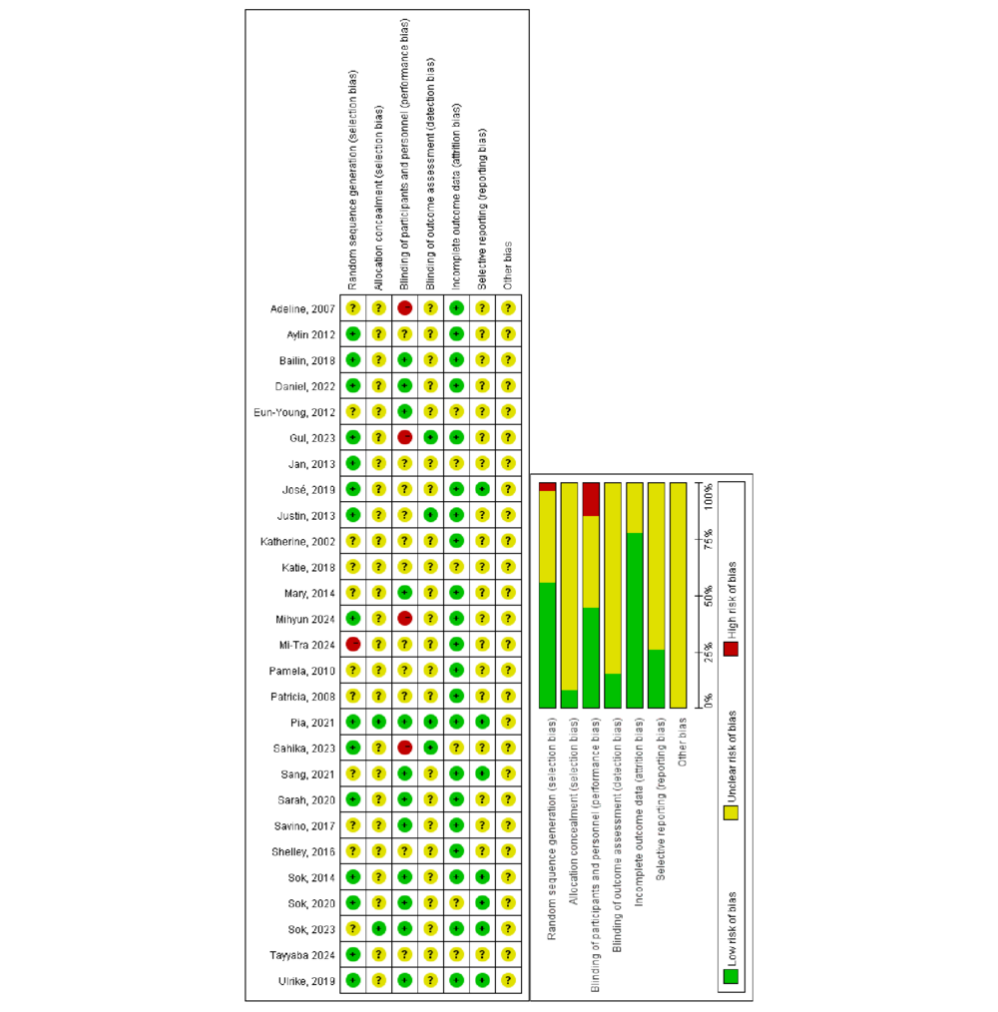
Risk of bias summary [16,18,30-54]. +: low risk of bias; –: high risk of bias; ?: unclear risk of bias.

Following Cochrane guidance [25], the risk of bias was assessed for all the included studies. The results are summarized in Figure 2 [16,18,30-54]. Out of 27 studies, 15 studies described an adequate random sequence generation method; 11 studies did not provide a clear description; and the remaining 1 study allocated participants to intervention and comparison arms based on the order in which they volunteered. Allocation concealment was not explicitly mentioned in most studies except 2. Blinding participants was impossible to avoid in this type of research but should not raise a major concern. Four studies had high-risk performance bias since researchers were not blinded and might give biased ratings; 12 studies were low-risk due to blinding of personnel; the others did not provide a clear description. The risk of detection bias was rated as unclear in most studies except 4, which clearly stated that statistical analysts were blinded. Most studies had low-risk attrition bias because no participants were lost, while participant dropout in 6 studies had an unclear impact on outcome assessment. Due to the absence of protocols, most studies had unclear reporting bias; 7 studies with protocols provided were rated as low risk. Given the difficulty of evaluating other biases (eg, volunteer bias), the risk was judged to be unclear for all studies.

### Effects of Interventions

#### Knowledge

**Figure 3:**
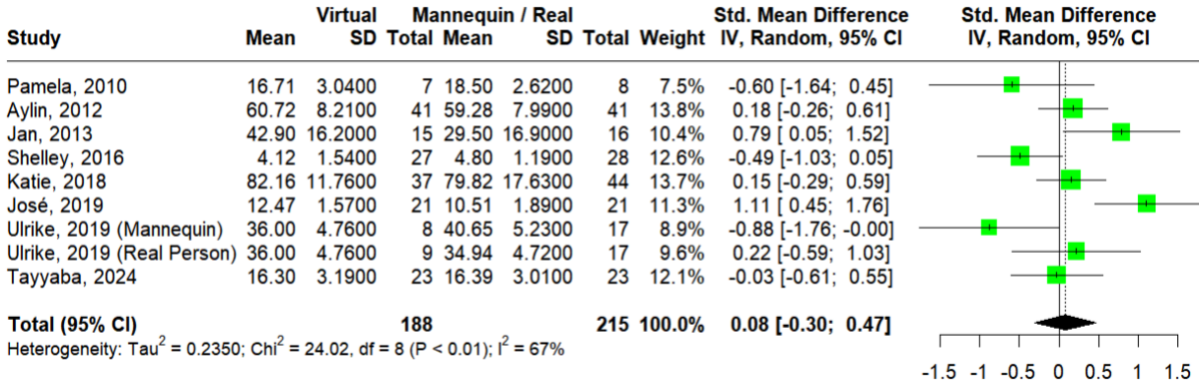
Forest plot of virtual simulations versus mannequins or real persons for knowledge [32,35,37,41,44-46,50].

A total of 8 studies assessed the outcome of knowledge [32,35,37,41,44-46,50]. The Egger test showed no statistically significant publication bias (*P*=.73). In Figure 3 [32,35,37,41,44-46,50], the pooled effect size did not reflect a significant difference between VSs and mannequins or RPs on the knowledge outcome (SMD=0.08; 95% CI –0.30 to 0.47; *I*^2^=67%; *P*=.002). TSA showed that the “inner wedge” area had been reached, indicating strong evidence that further studies would hardly be able to change the statistically insignificant result (Figure S1 in Multimedia Appendix 4). This result was consistent according to the leave-one-out analysis (Figure S2 in Multimedia Appendix 4 [32,35,37,41,44-46,50]).

For subgroup analysis by discipline (Figure S3a in Multimedia Appendix 4 [32,35,37,41,44-46,50]), 4 studies [32,35,37,41] and 4 studies [44-46,50] enrolled participants from medicine and nursing, respectively. Neither of these groups showed a significant difference between VSs and mannequins or RPs (SMD=–0.05; 95% CI –0.62 to 0.51; *I*^2^=59%; *P*=.04 for medicine and SMD=0.22; 95% CI –0.39 to 0.82; *I*^2^=78%; *P*<.001 for nursing), with no significant subgroup difference at *P*=.53.

For subgroup analysis by level (Figure S3b in Multimedia Appendix 4 [32,35,37,41,44-46,50]), 5 studies [35,44-46,50] and 3 studies [32,37,41] enrolled undergraduate and nonundergraduate participants, respectively. Neither of these groups showed a significant difference between VSs and mannequins or RPs (SMD=0.31; 95% CI –0.21 to 0.84; *I*^2^=75%; *P*<.001 for undergraduate and SMD=–0.25; 95% CI –0.71 to 0.22; *I*^2^=29%; *P*=.24 for nonundergraduate), with no significant subgroup difference at *P*=.12.

For subgroup analysis by comparison (Figure S3c in Multimedia Appendix 4 [32,35,37,41,44-46,50]), 2 studies [37,41] and 5 studies [35,44-46,50] included RP and mannequin arms, respectively; Weber et al [32] compared web-based simulators to both mannequins and RPs. Neither mannequins nor RPs was significantly different from VSs (SMD=0.15; 95% CI –0.40 to 0.71; *I*^2^=77%; *P*<.001 for mannequin and SMD=–0.06; 95% CI –0.48 to 0.37; *I*^2^=0%; *P*=.48 for RP), with no significant subgroup difference at *P*=.56.

Univariate meta-regression revealed that year of publication (β=.01; *P*=.92), age of participants (β=−.05; *P*=.29), nursing discipline (β=.27; *P*=.52), undergraduate level (β=.58; *P*=.14), and real person comparison (β=−.25; *P*=.58) had no effects on knowledge scores (Table S1 in Multimedia Appendix 4).

#### Procedural Skills

**Figure 4:**
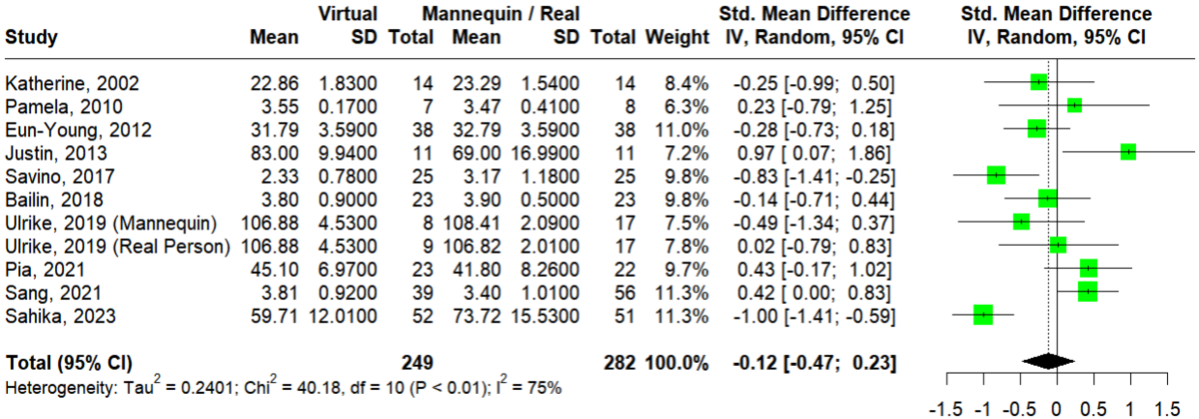
Forest plot of virtual simulations versus mannequins or real persons for procedural skills [16,18,32-34,36,37,42,49,51].

A total of 10 studies assessed the outcome of procedural skills [16,18,32-34,36,37,42,49,51]. The Egger test showed no statistically significant publication bias (*P*=.40). In Figure 4 [16,18,32-34,36,37,42,49,51], the pooled effect size did not reflect a significant difference between VSs and mannequins or RPs on the outcome of procedural skills (SMD=–0.12; 95% CI –0.47 to 0.23; *I*^2^=75%; *P*<.001). TSA showed that the RIS had been reached, implying a conclusive statistically insignificant result (Figure S4 in Multimedia Appendix 4). This result was consistent according to the leave-one-out analysis (Figure S5 in Multimedia Appendix 4 [16,18,32-34,36,37,42,49,51]).

For subgroup analysis by discipline (Figure S6a in Multimedia Appendix 4 [16,18,32-34,37,42,49,51]), 7 studies [16,18,32-34,36,37] and 3 studies [42,49,51] enrolled participants from medicine and nursing respectively. Only the nursing group showed a significant difference between VSs and mannequins or RPs (SMD=0.06; 95% CI –0.33 to 0.46; *I*^2^=64%; *P*<.001 for medicine and SMD=–0.55; 95% CI –1.07 to –0.03, *I*^2^=69%; *P*=.04 for nursing), with no significant subgroup difference at *P*=.07.

For subgroup analysis by level (Figure S6b in Multimedia Appendix 4 [16,18,32-34,36,37,42,49,51]), 3 studies [16,42,51] and 7 studies [18,32-34,36,37,49] enrolled undergraduate and nonundergraduate participants respectively. Neither of these groups showed a significant difference between VSs and mannequins or RPs (SMD=–0.29; 95% CI –1.10 to 0.52; *I*^2^=91%; *P*<.001 for undergraduate and SMD=–0.04; 95% CI –0.43 to 0.35; *I*^2^=56%; *P*=.03 for nonundergraduate), with no significant subgroup difference at *P*=.59.

For subgroup analysis by comparison (Figure S6c in Multimedia Appendix 4 [16,18,32-34,36,37,42,49,51]), 5 studies [33,34,36,49,51] and 4 studies [16,18,37,42] included mannequin and RP arms, respectively; Weber et al [32] compared web-based simulators to both mannequins and RPs. Neither mannequins nor RPs was significantly different from VSs (SMD=–0.22; 95% CI –0.63 to 0.19; *I*^2^=56%; *P*=.04 for mannequins and SMD=–0.00; 95% CI –0.60 to 0.59; *I*^2^=86%; *P*<.001 for RPs), with no significant subgroup difference at *P*=.57.

Univariate meta-regression revealed that year of publication (β=–.01; *P*=.71), age of participants (β=.05; *P*=.33), nursing discipline (β=–.60; *P*=.08), undergraduate level (β=–.25; *P*=.52), and real person comparison (β=.19; *P*=.61) had no effects on procedural skill scores (Table S1 in Multimedia Appendix 4).

#### Clinical Reasoning

**Figure 5:**
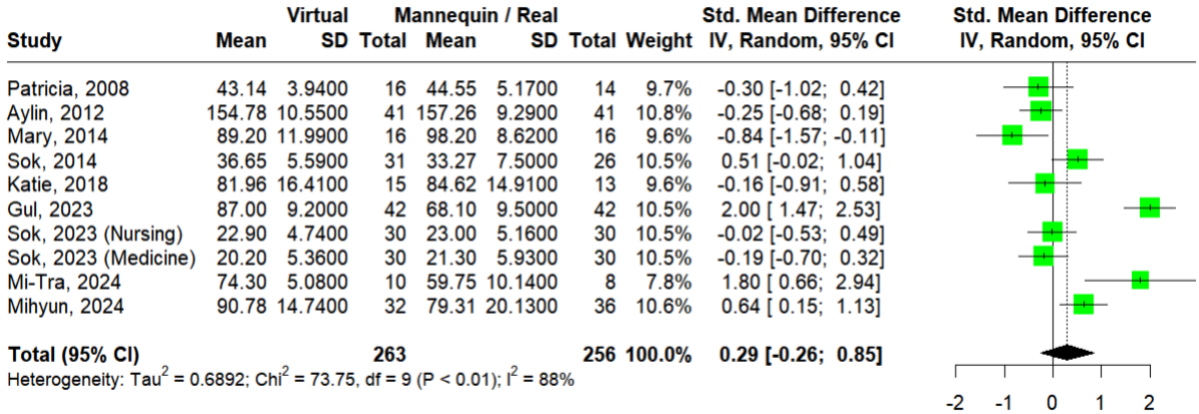
Forest plot of virtual simulations versus mannequins or real persons for clinical reasoning [38,40,43,45,47,48,50,52,53].

A total of 9 studies assessed the outcome of clinical reasoning [38,40,43,45,47,48,50,52,53]. The Egger test showed no statistically significant publication bias (*P*=.88). In Figure 5 [38,40,43,45,47,48,50,52,53], the pooled effect size did not reflect a significant difference between VSs and mannequins or RPs on the outcome of procedural skills (SMD=0.29; 95% CI –0.26 to 0.85; *I*^2^=88%; *P*<.01). TSA showed that the RIS had not been reached, implying further studies would be needed to verify the statistically insignificant result (Figure S7 in Multimedia Appendix 4). This result was consistent according to the leave-one-out analysis (Figure S8 in Multimedia Appendix 4).

For subgroup analysis by discipline (Figure S9a in Multimedia Appendix 4 [38,40,43,45,47,48,50,52,53]), 2 studies [38,40] and 6 studies [43,45,47,48,50,52] enrolled participants from medicine and nursing respectively. Liaw et al [53] enrolled participants from both fields. Neither of these groups showed a significant difference between VSs and mannequins or RPs (SMD=0.35; 95% CI –0.89 to 1.60; *I*^2^=81%; *P*<.001 for medicine and SMD=0.28; 95% CI –0.39 to 0.95; *I*^2^=90%; *P*<.001 for nursing), with no significant subgroup difference at *P*=.92.

For subgroup analysis by level (Figure S9b in Multimedia Appendix 4 [38,40,43,45,47,48,50,52,53]), 6 studies [43,45,47,50,52,53] and 2 studies [38,40] enrolled undergraduate and nonundergraduate participants, respectively; the study by Johnson et al [48] was excluded due to a mixed enrollment. Neither of these groups showed a significant difference between VSs and mannequins or RPs (SMD=0.36; 95% CI –0.23 to 0.96; *I*^2^=89%; *P*<.001 for undergraduate and SMD=0.70; 95% CI –1.35 to 2.75; *I*^2^=89%; *P*<.001 for nonundergraduate), with no significant subgroup difference at *P*=.76.

For subgroup analysis by comparison (Figure S9c in Multimedia Appendix 4 [38,40,43,45,47,48,50,52,53]), 6 studies [38,40,45,47,48,50] and 3 studies [43,52,53] included mannequin and RP arms, respectively. Neither mannequins nor RPs was significantly different from VSs (SMD=0.06; 95% CI –0.57 to 0.68; *I*^2^=75%; *P*<.001 for mannequins and SMD=0.60; 95% CI –0.37 to 1.58; *I*^2^=93%; *P*<.001 for RPs), with no significant subgroup difference at *P*=.35.

Univariate meta-regression revealed that year of publication (β=.08, *P*=.06), age of participants (β=–.02; *P*=.81), nursing discipline (β=–.05; *P*=.94), undergraduate level (β=–.27; *P*=.72), and real person comparison (β=.53; *P*=.35) had no effects on clinical reasoning scores (Table S1 in Multimedia Appendix 4).

#### Communication Skills

**Figure 6:**
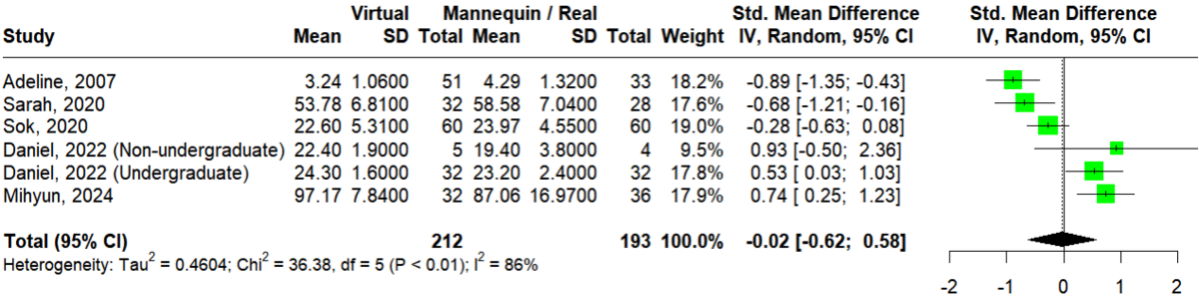
Forest plot of virtual simulations versus mannequins or real persons for communication skills [30,31,39,52,54].

A total of 5 studies assessed the outcome of communication skills [30,31,39,52,54]. The Egger test showed no statistically significant publication bias (*P*=.55). In Figure 6 [30,31,39,52,54], the pooled effect size did not reflect a significant difference between VSs and mannequins or RPs on the outcome of procedural skills (SMD=–0.02; 95% CI –0.62 to 0.58; *I*^2^=86%; *P*<.01). TSA showed that the “inner wedge” area had been reached, indicating strong evidence that further studies would hardly be able to change the statistically insignificant result (Figure S10 in Multimedia Appendix 4). This result was consistent according to the leave-one-out analysis (Figure S11 in Multimedia Appendix 4 [30,31,39,52,54]).

Since all 5 studies compared VSs to RPs, only subgroup analyses by discipline and level could be conducted. For subgroup analysis by discipline (Figure S12a in Multimedia Appendix 4 [30,31,39,52,54]), 3 studies [30,31,39] and 1 study [52] enrolled participants from medicine and nursing, respectively; and the study by Liaw et al [54] was excluded due to a mixed enrollment. Only the nursing group showed a significant difference between VSs and mannequins or RPs (SMD=–0.14; 95% CI –0.97 to 0.69; *I*^2^=86%; *P*<.001 for medicine and SMD=0.74; 95% CI 0.25-1.23 for nursing), with no significant subgroup difference at *P*=.07.

For subgroup analysis by level (Figure S12b in Multimedia Appendix 4 [30,31,39,52,54]), 4 studies enrolled undergraduate participants [31,39,52,54] and the study by Sapkaroski et al [30] enrolled both undergraduate and nonundergraduate participants. Neither of these groups showed a significant difference between VSs and mannequins or RPs (SMD=–0.12; 95% CI –0.75 to 0.51; *I*^2^=88%; *P*<.001 for undergraduate and SMD=0.93; 95% CI –0.50 to 2.36 for nonundergraduate), with no significant subgroup difference at *P*=.19.

Univariate meta-regression revealed that year of publication (β=.09; *P*=.02) had a positive effect on communication skill scores, whereas age of participants (β=–.07; *P*=.84), nursing discipline (β=.88; *P*=.33), undergraduate level (β=–1.05; *P*=.32), and real person comparison (β=.19; *P*=.61) had no effects (Table S1 in Multimedia Appendix 4). Since no studies compared VSs to mannequins, the effect of real person comparison could not be evaluated.

## Discussion

### Principal Findings

The aim of this meta-analysis was to evaluate the effectiveness of VSs in comparison with mannequins and RPs in medical and nursing education. We found that VSs and conventional simulations were not statistically different in teaching knowledge, procedural skills, clinical reasoning, and communication skills. Among nursing participants, VSs were more effective in improving procedural skills. For the training of communication skills, VSs tended to be increasingly effective with time.

This meta-analysis followed the original protocol of PROSPERO (CRD42023466622). TSA and meta-regression were added to further evaluate the accumulative evidence and identify potential sources of heterogeneity. We believe that these analyses do not negatively affect the reliability of our results; instead, they offer novel insights and strengthen the existing content. Here, we state the aforementioned deviations to ensure our study’s transparency.

### Comparison With Prior Work

There was no significant difference in knowledge gained between VSs and mannequins or RPs. This result aligns with a previous meta-analysis comparing 2D VSs with traditional education methods [21], while other analyses suggested that 3D VSs were more effective [22,55-57]. In our meta-analysis, almost all studies adopted 3D VSs, but the comparison was specifically narrowed down to conventional simulations. Our pooled effect size tended to favor VSs. Indeed, it can be beneficial to use VSs for knowledge delivery. First, digital platforms or software provide repeatable, information-rich feedback for learning [58]. Second, immersive technology (ie, devices that provide a sense of realism and immersion in the computer-generated world through sensory stimuli) such as VR promotes students’ interest with enhanced satisfaction, self-efficacy, and engagement [59]. Although VSs are viable alternatives to mannequins and RPs, undesirable usability may hinder the learning process. For instance, Cobbett and Snelgrove-Clarke [46] reported in their study that nearly half of the students expressed dislike for the VS due to technological issues such as the “online program was slow” and “didn't know where to find things.” Consequently, the development of VSs should consider features that enhance ease of use and users’ level of satisfaction (eg, step-by-step instructions, user-friendly interfaces) so that students allocate their time appropriately on learning instead of wrestling with the platform or software.

There was no significant difference in improving procedural skills between VSs and mannequins or RPs, while our pooled effect size tended to favor mannequins or RPs. Subgroup analyses showed that VSs were less effective among nursing participants. These results were inconsistent with previous meta-analyses comparing VSs with traditional education methods [21,22,57]. Such discrepancies may be due to different definitions of comparison. While VSs are advantageous for practicing and mastering skills relative to didactic teaching, this is not necessarily true if they are compared with mannequins and RPs. A gap exists between digital environments and real circumstances, and the abstraction of procedures may not be transferable to reality [60]. Notwithstanding, VSs are cost-effective alternatives that enable learners to repeat the training freely without worrying about time, space, and patient harm [61]. VSs may be designed to achieve better skill acquisition. First, the addition of haptic technology, for example, offers more realistic hands-on experiences [36]. Second, too much immersion may not be desirable as it can lead to cognitive overload and hamper procedure learning [42].

There was no significant difference in fostering clinical reasoning between VSs and mannequins or RPs. Again, this result does not conform to previous meta-analyses which compared VSs to no intervention or traditional education methods [21,62]. Our pooled effect size tended to favor VSs. It has been suggested that VSs, with a large number of customizable clinical scenarios, are highly suitable for improving clinical assessment and decision-making [63]. In particular, the following elements may be beneficial: a training duration of more than 30 minutes, varied clinical scenarios, nonimmersive 2D digital environments, and postscenario feedback [62]. Similar to procedural skills, nonimmersive VSs appear to be more effective than their immersive counterparts. According to the cognitive load theory. 3D digital environments can increase cognitive load as students get distracted by irrelevant stimuli [62]. More data are still required to reach a conclusive result regarding the comparative effectiveness of VSs in improving clinical reasoning.

There was no significant difference in enhancing communication skills between VSs and mannequins or RPs. This is in line with the findings of a previous meta-analysis comparing digital education to traditional learning [15]. Our pooled effect size tended to favor mannequins or RPs. It is noteworthy, however, that more recent studies tended to favor VSs. A possible explanation is that advances in technology can increasingly overcome the long-standing lack of realism and means for interaction in VSs [30]. Greater immersion can elicit a greater sense of “being there” for users [64]. This psychological experience is critical for communication training, to make learners feel as if they are having face-to-face conversations with digital avatars so that higher levels of empathy can be induced. Moreover, VSs have fewer limits on time or place, eliminate the costs of SP training, and offer a variety of clinical scenarios available for student communication training. These properties render VSs with great potential as novel methods for teaching communication skills.

### Strengths and Limitations

To the best of our knowledge, this is the first meta-analysis comparing the effectiveness of VSs and conventional simulations. Strengths of this study include pragmatic research questions, well-defined outcome measures, comprehensive searches of the most up-to-date RCTs (ie, the gold standard for evaluating the effectiveness of interventions), and critical analyses of the evidence. Our results indicate that VSs are viable alternatives to face-to-face learning modalities. For institutions, time and money saved can be invested in other research and teaching projects. For students, the removal of time, space, and location limitations leads to greater freedom of learning and wider popularization of education.

On the other hand, limitations must be acknowledged when interpreting the results of this study. First, researchers in several studies were not blinded and might introduce bias when assessing participants. Second, certain subgroup or meta-regression analyses were not feasible or included only one entry due to the limited number of studies. Further analyses may be possible when more RCTs are published. Third, the heterogeneity of the included studies could not be fully explained by leave-one-out, subgroup, or meta-regression analyses. This lack of explanation may be attributed to other unspecified sources of methodological and subject heterogeneity.

### Conclusions

Overall, this meta-analysis did not find significant differences between VSs and mannequins or RPs in improving knowledge, procedural skills, clinical reasoning, and communication skills. For procedural skill acquisition, VSs were less effective than mannequins or RPs among nursing participants. For communication skill development, VSs exhibited increasing potency with more advanced technology over time. Given other prominent advantages of VSs (eg, cost-effectiveness, flexibility regarding time or space), it is worth considering these tools as alternatives to traditional education methods. As technology continues to evolve, the comparative effectiveness of VSs will need to be reevaluated and updated. In particular, it is anticipated to witness the integration of emerging large language models (eg, ChatGPT) in VSs and see if they can revolutionize educational practices. We also recommend future research to investigate the effectiveness of VSs across various design configurations.

## Appendix

Multimedia Appendix 1 PRISMA (Preferred Reporting Items for Systematic Reviews and Meta-Analyses) checklist.

Multimedia Appendix 2 Search strategies.

Multimedia Appendix 3 Characteristics of selected 27 randomized controlled trials.

Multimedia Appendix 4 Trial sequential, leave-one-out, subgroup and meta-regression analyses.

## Abbreviations

AI: artificial intelligence
PRISMA: Preferred Reporting Items for Systematic Reviews and Meta-analyses
RCT: randomized controlled trial
RIS: required information size
RP: real person
SMD: standardized mean difference
SP: standardized patient
TSA: trial sequential analysis
VP: virtual patient
VR: virtual reality
VS: virtual simulation
