# Supplementary material for: Effectiveness of Virtual Simulations Versus Mannequins and Real Persons in Medical and Nursing Education: Meta-Analysis and Trial Sequential Analysis of Randomized Controlled Trials
Source: J Med Internet Res. 2024 Dec 5;26:e56195. doi: 10.2196/56195 (PMC11659697; doi:10.2196/56195)
Supplement: Multimedia Appendix 2 [file jmir_v26i1e56195_app2.docx]

## **Multimedia Appendix 2**

## Search strategies

PubMed

#1. (“virtual patients”[Title/Abstract] OR “virtual patient”[Title/Abstract] OR “digital patients”[Title/Abstract] OR “digital patient”[Title/Abstract] OR avatar*[Title/Abstract] OR “virtual standardized patients”[Title/Abstract] OR “virtual standardized patient”[Title/Abstract] OR “virtual simulation”[Title/Abstract] OR “virtual simulations”[Title/Abstract] OR “virtual reality”[Title/Abstract] OR “Virtual Reality”[MeSH Terms] OR “artificial intelligence”[Title/Abstract] OR “Artificial Intelligence”[MeSH Terms] OR “computer simulation”[Title/Abstract] OR “Computer Simulation”[MeSH Terms] OR “e-learning”[Title/Abstract] OR “digital learning”[Title/Abstract] OR “online learning”[Title/Abstract] OR “Education, Distance”[MeSH Terms])

#2. (“standardized patients”[Title/Abstract] OR “standardized patient”[Title/Abstract] OR “standardised patients”[Title/Abstract] OR “standardised patient”[Title/Abstract] OR “simulated patients”[Title/Abstract] OR “standard patient”[Title/Abstract] OR “standard patients”[Title/Abstract] OR “simulated patient”[Title/Abstract] OR “human patient”[Title/Abstract] OR “human patients”[Title/Abstract] OR “real patient”[Title/Abstract] OR “real patients”[Title/Abstract] OR “role-play”[Title/Abstract] OR “healthy volunteers”[Title/Abstract] OR manikin*[Title/Abstract] OR mannequin*[Title/Abstract] OR “Manikins”[MeSH Terms])

#3. (“medical students”[Title/Abstract] OR “Students, Medical”[MeSH Terms] OR “nursing students”[Title/Abstract] OR “Students, Nursing”[MeSH Terms] OR “nurses”[Title/Abstract] OR “Nurses”[MeSH Terms] OR “medical education”[Title/Abstract] OR “Education, Medical”[MeSH Terms] OR “nursing education”[Title/Abstract] OR “Education, Nursing”[MeSH Terms] OR

resident*[Title/Abstract] OR physician*[Title/Abstract] OR “Physicians”[MeSH Terms] OR clinician*[Title/Abstract] OR practitioner*[Title/Abstract] OR “interns”[Title/Abstract] OR “intern”[Title/Abstract] OR trainee*[Title/Abstract] OR “Education, Medical, Continuing”[MeSH Terms] OR “continuing medical education”[Title/Abstract])

#4. #1 AND #2 AND #3

Embase

#1. (“virtual patients”:ti,ab,kw OR “virtual patient”:ti,ab,kw OR “digital patients”:ti,ab,kw OR “digital patient”:ti,ab,kw OR avatar*:ti,ab,kw OR “virtual standardized patients”:ti,ab,kw OR “virtual standardized patient”:ti,ab,kw OR “virtual simulation”:ti,ab,kw OR “virtual simulations”:ti,ab,kw OR “virtual reality”:ti,ab,kw OR “artificial intelligence”:ti,ab,kw OR “computer simulation”:ti,ab,kw OR “e-learning”:ti,ab,kw OR “digital learning”:ti,ab,kw OR “online learning”:ti,ab,kw)

#2. (“standardized patients”:ti,ab,kw OR “standardized patient”:ti,ab,kw OR “standardised patients”:ti,ab,kw OR “standardised patient”:ti,ab,kw OR “simulated patients”:ti,ab,kw OR “standard patient”:ti,ab,kw OR “standard patients”:ti,ab,kw OR “simulated patient”:ti,ab,kw OR “human patient”:ti,ab,kw OR “human patients”:ti,ab,kw OR “real patient”:ti,ab,kw OR “real patients”:ti,ab,kw OR “role-play”:ti,ab,kw OR “healthy volunteers”:ti,ab,kw OR manikin*:ti,ab,kw OR mannequin*:ti,ab,kw)

#3. (“medical students”:ti,ab,kw OR “nursing students”:ti,ab,kw OR “nurses”:ti,ab,kw OR “medical education”:ti,ab,kw OR “nursing education”:ti,ab,kw OR resident*:ti,ab,kw OR physician*:ti,ab,kw OR clinician*:ti,ab,kw OR practitioner*:ti,ab,kw OR “interns”:ti,ab,kw OR “intern”:ti,ab,kw OR trainee*:ti,ab,kw OR “continuing medical education”:ti,ab,kw)

#4. #1 AND #2 AND #3

The Cochrane Library

#1. (“virtual patients” OR “virtual patient” OR “digital patients” OR “digital patient” OR avatar* OR “virtual standardized patients” OR “virtual standardized patient” OR “virtual simulation” OR “virtual simulations” OR “virtual reality” OR “artificial intelligence” OR “computer simulation” OR “e-learning” OR “digital learning” OR “online learning”)

#2. (“standardized patients” OR “standardized patient” OR “standardised patients” OR “standardised patient” OR “simulated patients” OR “standard patient” OR “standard patients” OR “simulated patient” OR “human patient” OR “human patients” OR “real patient” OR “real patients” OR “role-play” OR “healthy volunteers” OR manikin* OR mannequin*)

#3. (“medical students” OR “nursing students” OR “nurses” OR “medical education” OR “nursing education” OR resident* OR physician* OR clinician* OR practitioner* OR “interns” OR “intern” OR trainee* OR “continuing medical education”)

#4. #1 AND #2 AND #3

ERIC

#1. (abstract:"virtual patient" OR abstract:"digital patient" OR abstract:"avatar" or abstract:"virtual standardized patient" OR abstract:"virtual simulation" OR abstract:"virtual reality" OR abstract:"artificial intelligence" OR abstract:"computer simulation" OR abstract:"e-learning" OR abstract:"digital learning" OR abstract:"online learning")

#2. (abstract:"standardized patient" OR abstract:"standardised patient" OR abstract:"standard patient" OR abstract:"simulated patient" OR abstract:"human patient" OR abstract:"real patient" OR abstract:"role-play" OR abstract:"healthy volunteer" OR abstract:"manikin" OR abstract:"mannequin")

#3. (abstract:"medical student" OR abstract:"nursing student" OR abstract:"nurse" OR abstract:"medical education" OR abstract:"nursing education" OR abstract:"resident" OR abstract:"physician" OR abstract:"clinician" or abstract:"practitioner" OR abstract:"intern" OR abstract:"trainee" OR abstract:"continuing medical education")

#4. #1 AND #2 AND #3
