# Supplementary material for: Effectiveness of Virtual Simulations Versus Mannequins and Real Persons in Medical and Nursing Education: Meta-Analysis and Trial Sequential Analysis of Randomized Controlled Trials
Source: J Med Internet Res. 2024 Dec 5;26:e56195. doi: 10.2196/56195 (PMC11659697; doi:10.2196/56195)
Supplement: Multimedia Appendix 3 [file jmir_v26i1e56195_app3.docx]

## Characteristics of selected 27 randomized controlled trials

| **Study** | **Country** | **Teaching subject** | **Discipline** | **Level** | **Age [Mean/median (SD/range)]** | **Intervention** | **Comparison** | **Outcome measurement** | **Main results** |
| --- | --- | --- | --- | --- | --- | --- | --- | --- | --- |
| Mi-Tra et al [43], 2024 | United Kingdom | Assessment and management of acute surgical scenarios | Medicine | Non-undergraduate (junior doctors) | 26.0 (2.2) | Oxford Medical Simulation software application with an immersive VR headset (n=10) | Mannequin (n=8) | Clinical reasoning  - Intervention: rated by the application  - Comparison: rated by assessors according to a simulation marking scheme following the application | Performance scores in intervention vs comparison: 74.30 ± 5.08 vs 59.75 ± 10.14 (P=0.04) |
| Mihyun et al [55], 2024 | Korea | Psychiatric nursing | Nursing | Undergraduate | 24.5 (Not reported) | Immersive VR simulation program (n=32) | Role-play (n=36) | Clinical reasoning  - Intervention: rated by assessors using mental health nursing performance checklists  - Comparison: same as above  Communication skills  - Intervention: rated by assessors using mental health nursing performance checklists  - Comparison: same as above | For clinical reasoning, performance scores in intervention vs comparison: 90.78 ± 14.74 vs 79.31 ± 20.13 (P=0.011)  For communication skills, performance scores in intervention vs comparison: 97.17 ± 7.84 vs 87.06 ± 16.97 (P=0.003) |
| Tayyab et al [44], 2024 | Pakistan | Clinical reasoning skills | Medicine | Non-undergraduate (residents) | 28.5 (3.0) | VR simulation selected from the “American Academy of Ophthalmology” website (n=23) | Real SP simulation (n=23) | Knowledge  - Intervention: rated by a knowledge assessment test with multiple-choice questions  - Comparison: same as above | Performance scores in intervention vs comparison: 16.30 ± 3.19 vs 16.39 ± 3.01 (P not directly reported) |
| Sahika et al [45], 2023 | Türkiye | Breast self-examination | Nursing | Undergraduate | 19.6 (1.2) | AI Assisted Interactive Screen-Based Simulation application (n=52) | Real SP simulation (n=51) | Procedural skills  - Intervention: rated by the application according to a breast self-examination checklist algorithm  - Comparison: rated by assessors based on the breast self-examination checklist answer key | Performance scores in intervention vs comparison: 59.71 ± 12.01 vs 73.72 ± 15.53 (P<0.001) |
| Gul et al [46], 2023 | Türkiye | Clinical decision-making | Nursing | Undergraduate | 21.2 (Not reported) | Body Interact, a three-dimensional computer-based  program (n=42) | CAE Juno simulator, a realistic, human-sized  model (n=42) | Clinical reasoning  - Intervention: rated by the program according to the priority of interventions made by students  - Comparison: rated by assessors using performance assessment forms, which were designed to be the same as the program’s performance reports | Performance scores in intervention vs comparison: 87.00 ± 9.20 vs 68.10 ± 9.50 (P<0.001) |
| Sok et al [56], 2023 | Singapore | Clinical deterioration | Medicine  Nursing | Undergraduate | 21.7 (1.0)  22.1 (1.0) | Desktop VR platform (n=60) | Real SP simulation (n=60) | Clinical reasoning  - Intervention: rated by assessors using the modified Rescuing A Patient In Deteriorating Situations tool  - Comparison: same as above | For medical participants, performance scores in intervention vs comparison: 20.20 ± 5.36 vs 21.30 ± 5.93 (P=0.45)  For nursing participants, performance scores in intervention vs comparison: 22.90 ± 4.74 vs 23.00 ± 5.16 (P=0.94) |
| Daniel et al [33], 2022 | Australia | Clinical communication skills | Medicine | Non-undergraduate (clinicians)  Undergraduate | Not reported  Not reported | Clinical Education Training Solution VR Clinic software (n=37) | Role-play (n=36) | Communication skills  - Intervention: rated based on assessments performed by the expert panel of designers  - Comparison: same as above | For clinicians, performance scores in intervention vs comparison: 22.40 ± 1.90 vs 19.40 ± 3.80 (P=0.17)  For undergraduate students, performance scores in intervention vs comparison: 24.30 ± 1.60 vs 23.20 ± 2.40 (P<0.05) |
| Sang et al [16], 2021 | Korea | Neurological examination | Medicine | Undergraduate | 25.1 (1.9) | VR-based neurologic examination teaching tool (n=39) | Real SP simulation (n=56) | Procedural skills  - Intervention: rated by assessors using the Neurologic Physical Exam score  - Comparison: same as above | Performance scores in intervention vs comparison: 3.81 ± 0.92 vs 3.40 ± 1.01 (P=0.043) |
| Pia et al [18], 2021 | Denmark | Thoracic ultrasound skills | Medicine | Non-undergraduate (physicians) | 30.8 (Not reported) | US Mentor Simulator, a VR ultrasound simulator (n=23) | Healthy volunteer (n=22) | Procedural skills  - Intervention: rated by assessors using the lung ultrasound objective  structured assessment of ultrasound skills score  - Comparison: same as above | Performance scores in intervention vs comparison: 45.1 (95% CI 42.2–47.9) vs 41.8 (95% CI 38.4–45.3) (P=0.384) |
| Sok et al [57], 2020 | Singapore | Team communication skills | Medicine  Nursing | Undergraduate | 22.2 (2.1) | Create Real-time Experience and Teamwork in Virtual Environment, a computer-based VR simulation (n=60) | Real SP simulation (n=60) | Communication skills  - Intervention: rated by assessors using a validated team communication scale  - Comparison: same as above | Performance scores in intervention vs comparison: 22.60 ± 5.31 vs 23.97 ± 4.55 (P=0.29) |
| Sarah et al [34], 2020 | United States | Clinical communication skills | Medicine | Undergraduate | 26.3 (1.7) | Three-dimensional VP simulation (n=32) | Real SP simulation (n=28) | Communication skills  - Intervention: rated by assessors using the modified Standardized Patient Checklist and Rating Scale  - Comparison: same as above | Performance scores in intervention vs comparison: 53.78 ± 6.81 vs 58.58 ± 7.04 (P=0.253) |
| José et al [47], 2019 | Portugal | Clinical scenario knowledge | Nursing | Undergraduate | 19.9 (2.0) | Body Interact, a three-dimensional computer-based  program (n=21) | Low-fidelity mannequin (n=21) | Knowledge  - Intervention: rated by a knowledge assessment test with true or false and multiple-choice questions  - Comparison: same as above | Performance scores in intervention vs comparison: 12.47 ± 1.51 vs 10.51 ± 1.89 (P=0.001) |
| Ulrike et al [35], 2019 | Australia | Basic perioperative transesophageal echocardiography | Medicine | Non-undergraduate (residents) | 29.5 (Not reported)  29.9 (Not reported) | Three-dimensional online simulator (n=17) | 1. CAE Vimedix Simulator, a simulation mannequin (n=17) 2. Real patient simulation (n=17) | Procedural skills  - Intervention: rated by assessors using a grading scale applied successfully in previous investigations  - Comparison: same as above  Knowledge  - Intervention: rated by a knowledge assessment test with multiple-choice questions  - Comparison: same as above | For procedural skills, performance scores in intervention vs comparison 1 vs comparison 2: 106.88 ± 4.53 vs 108.41 ± 2.09 vs 106.82 ± 2.01 (P=0.022)  For knowledge, performance scores in intervention vs comparison 1 vs comparison 2: 36.00 ± 4.76 vs 40.65 ± 5.23 vs 34.94 ± 4.72 (P=0.005) |
| Bailin et al [36], 2018 | China | Fiberoptic bronchoscope manipulation | Medicine | Non-undergraduate (residents) | 25.1 (Not reported) | GI-Bronch Mentor, a VR simulator (n=23) | High-fidelity Mannequin (n=23) | Procedural skills  - Intervention: rated by assessors using a validated global rating scale of fiberoptic bronchoscope manipulation  - Comparison: same as above | Performance scores in intervention vs comparison: 3.8 ± 0.9 vs 3.9 ± 0.5 (P not directly reported) |
| Katie [48], 2018 | United States | Chronic obstructive pulmonary disease exacerbation | Nursing | Undergraduate | 32.9 (8.5) | vSim, a three-dimensional VS program (n=15) | Mannequin (n=13) | Knowledge  - Intervention: rated by a knowledge assessment test with multiple-choice questions  - Comparison: same as above  Clinical reasoning  - Intervention: rated by assessors using the Creighton Simulation Evaluation Instrument  - Comparison: same as above | For knowledge, performance scores in intervention vs comparison: 82.16 ± 11.76 vs 79.82 ± 17.63 (P=0.476)  For clinical reasoning, performance scores in intervention vs comparison: 81.96 ± 16.41 vs 84.62 ± 14.91 (P=0.660) |
| Savino et al [37], 2017 | Italy | Mechanical ventilation | Medicine | Non-undergraduate (residents) | 29.3 (Not reported) | Virtual MV v4.0 (Mechanical Ventilation Simulator), a two-dimensional online software program (n=25) | Mannequin (n=25) | Procedural skills  - Intervention: rated by assessors using the Creighton Simulation Evaluation Instrument  - Comparison: same as above | Performance scores in intervention vs comparison: 2.0 (2.0-3.0) vs 3.0 (2.5-4.0) (P=0.005) |
| Shelley et al [49], 2016 | Canada | Maternal-newborn nursing | Nursing | Undergraduate | 25.0 (Not reported) | vSim, a three-dimensional VS program (n=27) | High-fidelity  mannequin (n=28) | Knowledge  - Intervention: rated by a knowledge assessment test with multiple-choice questions  - Comparison: same as above | Performance scores in intervention vs comparison: 4.12 ± 1.54 vs 4.80 ± 1.19 (P=0.09) |
| Sok et al [50], 2014 | Singapore | Clinical deterioration | Nursing | Undergraduate | 21.9 (1.1) | e-RAPIDS (Rescuing a Patient in Deteriorating Situations), a three-dimensional VP simulation (n=31) | Mannequin (n=26) | Clinical reasoning  - Intervention: rated by assessors using a RAPIDS tool  - Comparison: same as above | Performance scores in intervention vs comparison: 36.65 ± 5.59 vs 33.27 ± 7.50 (P=0.12) |
| Mary et al [51], 2014 | United States | Critical care | Nursing | Non-undergraduate (graduate students) | Not reported | DXR Clinician, a two-dimensional Web-based software-training program (n=16) | SimMan 3G and SimMan Essentials, full body mannequins (n=16) | Clinical reasoning  - Intervention: rated by assessors using a validated performance checklist  - Comparison: same as above | Performance scores in intervention vs comparison: 89.20 ± 11.99 vs 98.20 ± 8.62 (P=0.02) |
| Jan et al [38], 2013 | Sweden | Image quality assessment | Medicine | Undergraduate | 26.5 (Not reported) | Three-dimensional Virtual radiography simulator (n=15) | Mannequin (n=16) | Knowledge  - Intervention: rated by a knowledge assessment test with multiple-choice questions  - Comparison: same as above | Performance scores in intervention vs comparison: 42.9 ± 16.2 vs 29.5 ± 16.9 (P not directly reported) |
| Justin et al [39], 2013 | Canada | Surgical fixation of an ulnar fracture | Medicine | Non-undergraduate (residents) | Not reported | VR simulator with haptics (n=11) | Sawbones simulator model (n=11) | Procedural skills  - Intervention: rated by assessors using a global rating scale  - Comparison: same as above | Performance scores in intervention vs comparison: 83.00 ± 9.94 vs 69.00 ± 16.99 (P<0.05) |
| Aylin et al [53], 2012 | Türkiye | Preoperative and  postoperative care  management | Nursing | Undergraduate | 20.5 (1.1) | Two-dimensional screen-based computer simulator (n=41) | Mannequin (n=41) | Knowledge  - Intervention: rated by a knowledge assessment test with multiple-choice questions  - Comparison: same as above  Clinical reasoning  - Intervention: rated by assessors using the Clinical Decision Making in Nursing Scale  - Comparison: same as above | For knowledge, performance scores in intervention vs comparison: 60.72 ± 8.21 vs 59.28 ± 7.99 (P=0.421)  For clinical reasoning, performance scores in intervention vs comparison: 154.78 ± 10.55 vs 157.26 ± 9.29 (P=0.065) |
| Eun-Young et al [54], 2012 | Korea | Venipuncture | Nursing | Undergraduate | 20.2 (Not reported) | ARSim IV-100, a VR simulator with haptics (n=38) | Arm model (n=38) | Procedural skills  - Intervention: rated by assessors according to the intravenous injection protocol  - Comparison: same as above | Performance scores in intervention vs comparison: 31.79 ± 3.59 vs 32.79 ± 3.58 (P not directly reported) |
| Pamela et al [40], 2010 | United States | Disaster triage | Medicine | Non-undergraduate (residents) | Not reported | CAVE, a full-immersion VR simulator (n=7) | Real SP simulation (n=8) | Procedural skills  - Intervention: rated by assessors using a triage assessment instrument  - Comparison: same as above  Knowledge  - Intervention: rated by a knowledge assessment test with multiple-choice questions  - Comparison: same as above | For procedural skills, performance scores in intervention vs comparison: 3.55 ± 0.17 vs 3.47 ± 0.41 (P not directly reported)  For knowledge, performance scores in intervention vs comparison: 16.71 ± 3.04 vs 18.50 ± 2.62 (P not directly reported) |
| Patricia et al [41], 2008 | United States | Crisis management | Medicine | Non-undergraduate (graduate students) | Not reported | Three-dimensional software program (n=16) | Mannequin (n=14) | Clinical reasoning  - Intervention: rated by assessors using the Emergency Medicine Crisis Resource Management scale  - Comparison: same as above | Performance scores in intervention vs  comparison: 43.14 ± 3.94 vs 44.55 ± 5.17 (P=0.40) |
| Adeline et al [42], 2007 | United States | Complex communication skills (e.g., nonverbal behaviors and empathy) | Medicine | Undergraduate | Not reported | Three-dimensional VP simulation (n=51) | Real SP simulation (n=33) | Communication skills  - Intervention: rated by assessors using a Likert-type scale with  anchored descriptors Management scale  - Comparison: same as above | Performance scores in intervention vs comparison: 3.24 ± 1.06 vs 4.29 ± 1.32 (P<0.05) |
| Katherine et al [52], 2002 | Hong Kong | Intravenous cannulation | Nursing | Non-undergraduate (nurses) | Not reported | CathSim Intravenous Training System, a VR simulator (n=14) | Mannequin (n=14) | Procedural skills  - Intervention: rated by assessors using an intravenous cannulation checklist  - Comparison: same as above | Performance scores in intervention vs comparison: 22.86 ± 1.83 vs 23.29 ± 1.54 (P=0.509) |

Mean ± standard deviation. Median (interquartile range).

AI=artificial intelligence. SD=standard deviation. SP=standardized patient. VP=virtual patient. VR=virtual reality. VS=virtual simulation.
