# Supplementary material for: Effectiveness of Virtual Simulations Versus Mannequins and Real Persons in Medical and Nursing Education: Meta-Analysis and Trial Sequential Analysis of Randomized Controlled Trials
Source: J Med Internet Res. 2024 Dec 5;26:e56195. doi: 10.2196/56195 (PMC11659697; doi:10.2196/56195)
Supplement: Multimedia Appendix 4 [file jmir_v26i1e56195_app4.docx]

## **Multimedia Appendix 4**

**Trial sequential, leave-one-out, subgroup and meta-regression analyses**

**Figure S1: Trial sequential analysis for knowledge**

**
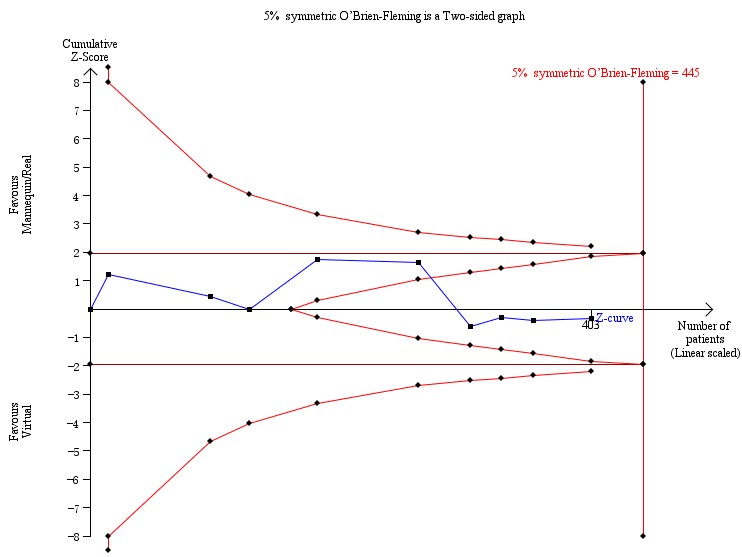
**

**Figure S2: Leave-one-out analysis for knowledge**


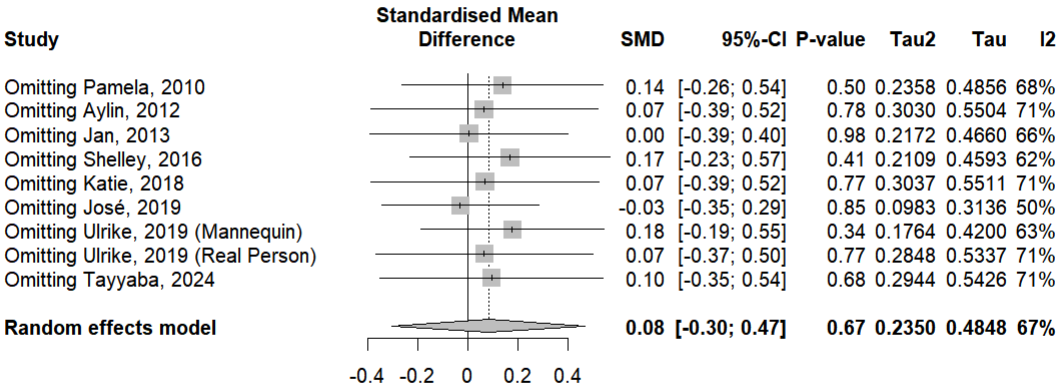


**Figure S3: Subgroup analyses for knowledge**

**a)**


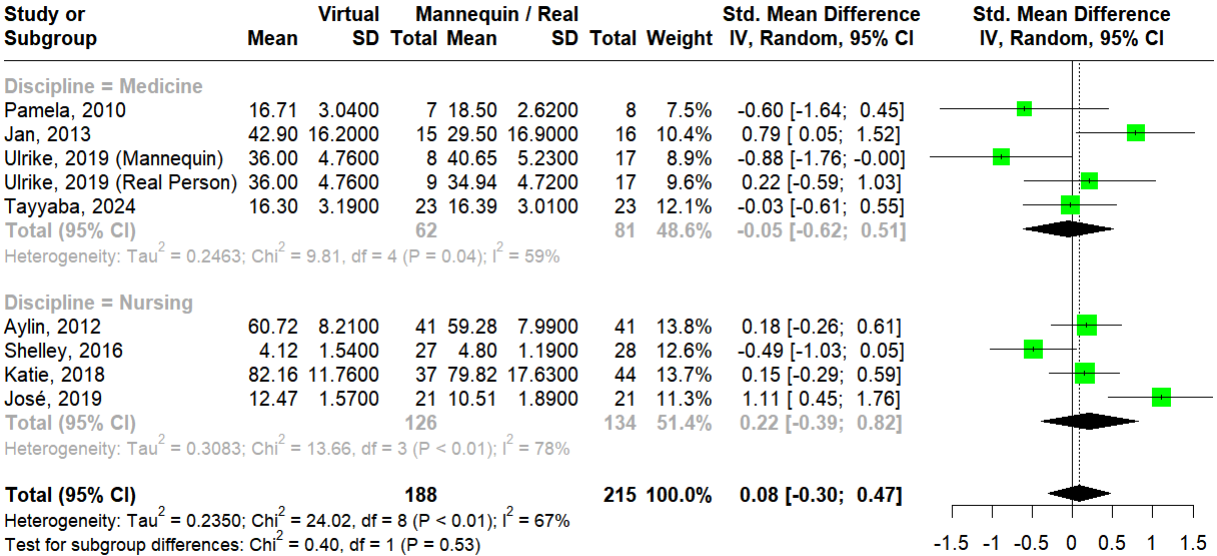


**b)**

**
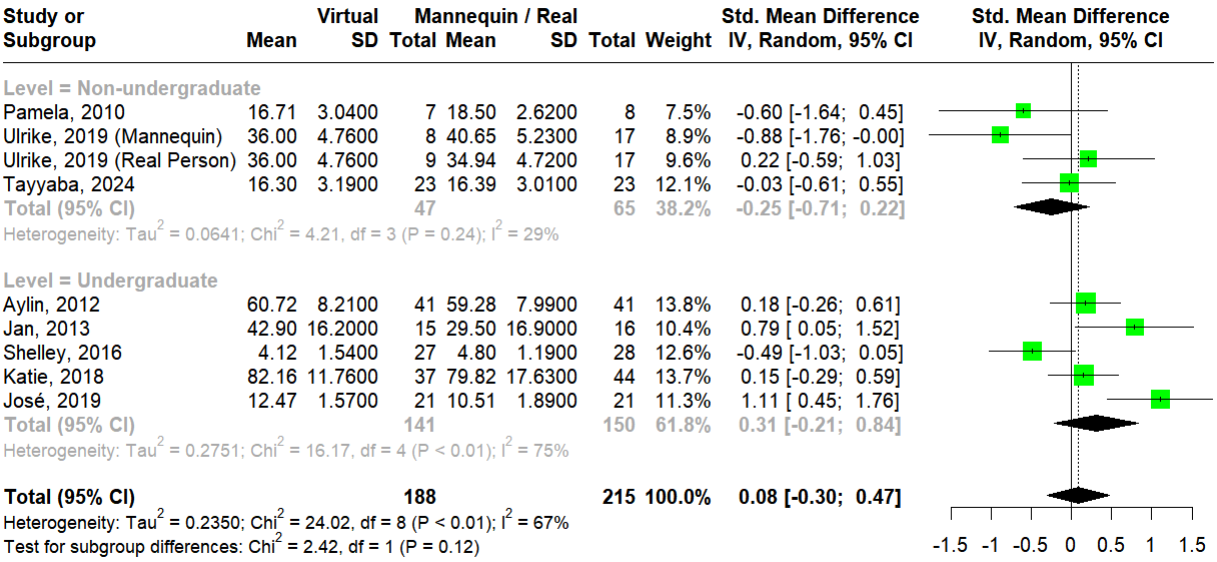
**

**c)**

**
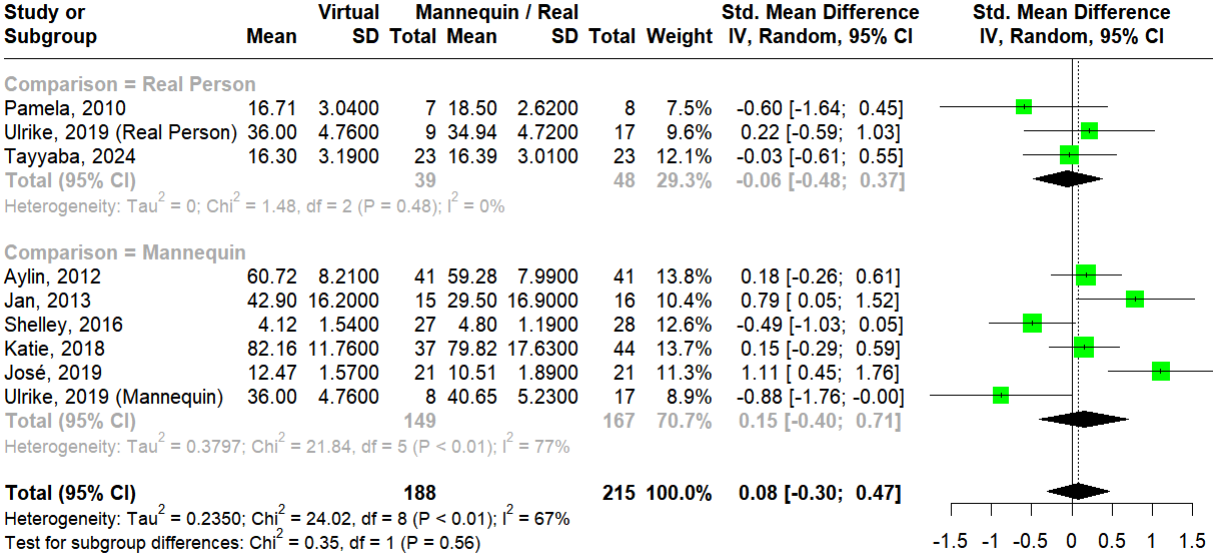
**

Subgroup analysis by a) discipline (medicine; nursing), b) level (undergraduate; non-undergraduate) and c) comparison (mannequin; real person).

**Figure S4: Trial sequential analysis for procedural skills**


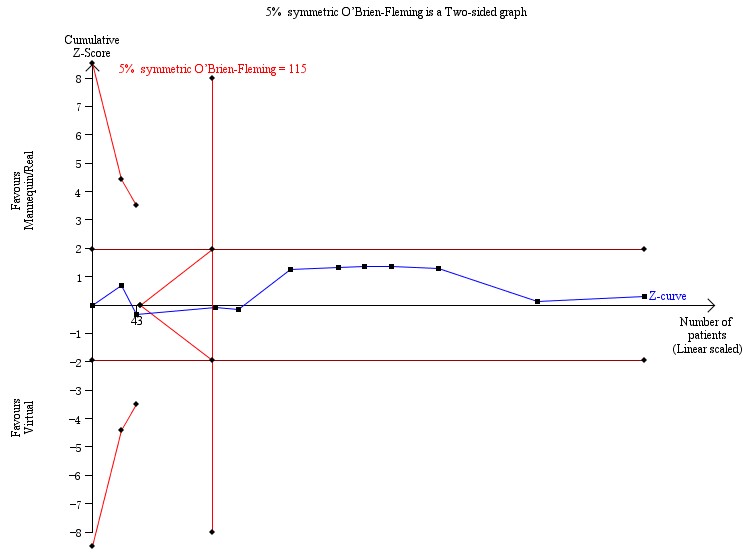


**Figure S5: Leave-one-out analysis for procedural skills**


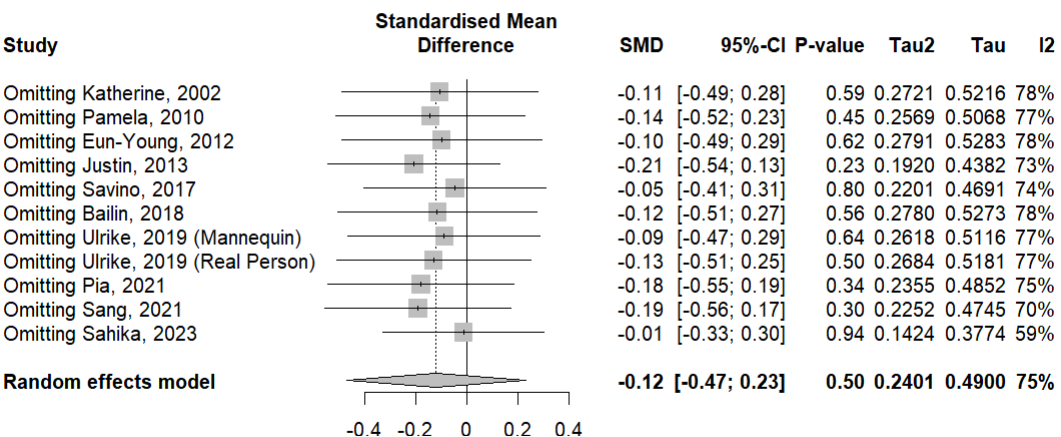


**Figure S6: Subgroup analyses for procedural skills**

**a)**


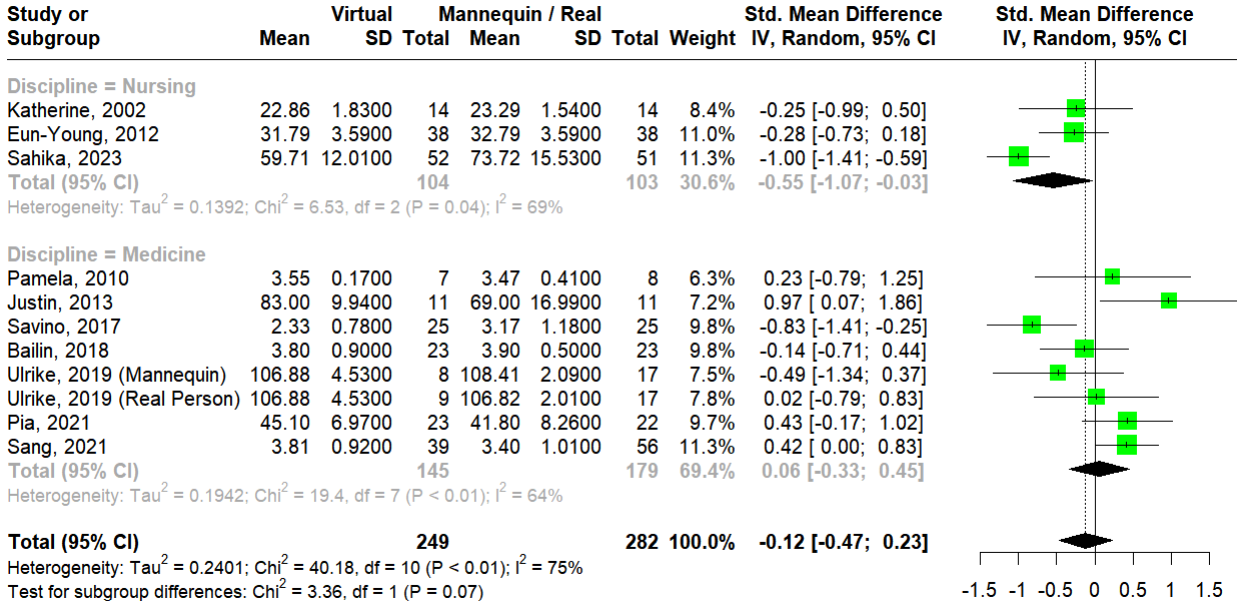


**b)**

**
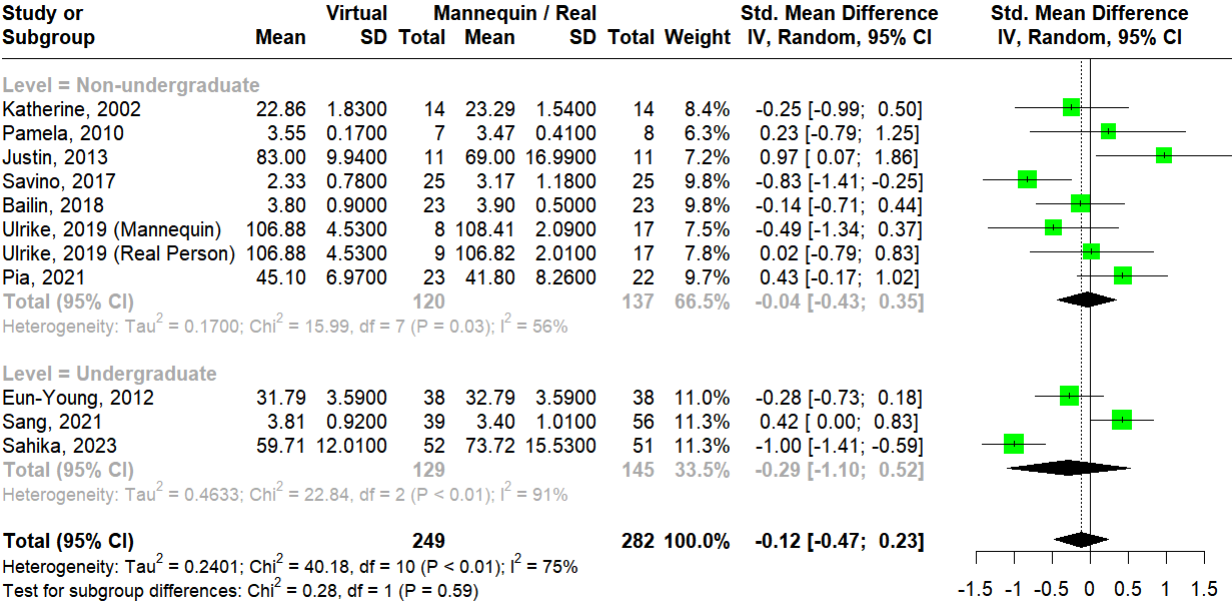
**

**c)**

**
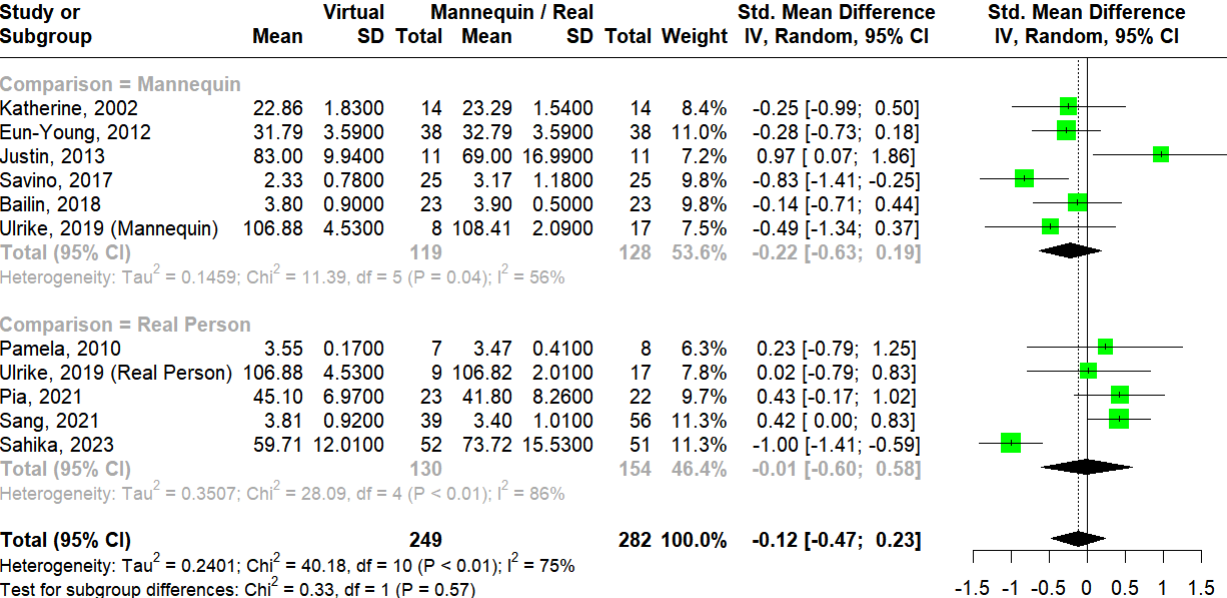
**

Subgroup analysis by a) discipline (medicine; nursing), b) level (undergraduate; non-undergraduate) and c) comparison (mannequin; real person).

**Figure S7: Trial sequential analysis for clinical reasoning**

**
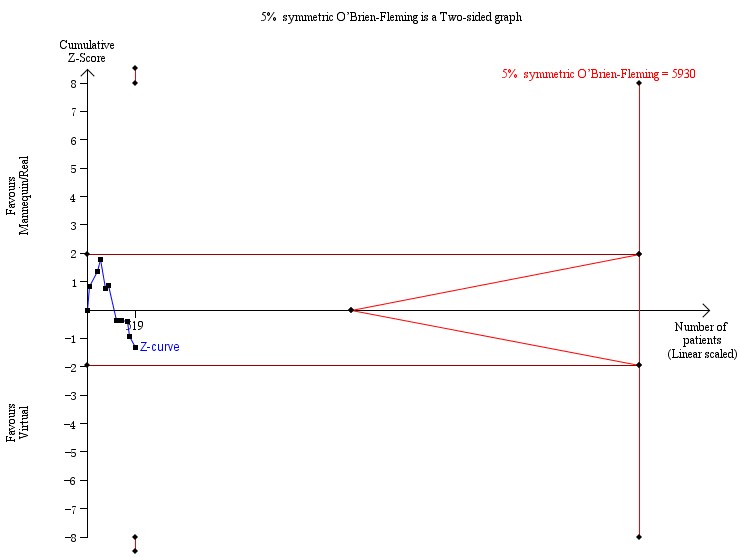
**

**Figure S8: Leave-one-out analysis for clinical reasoning**


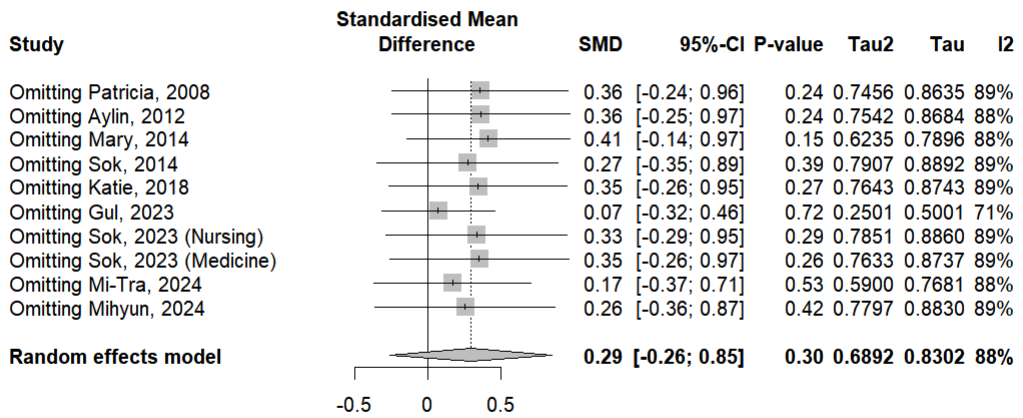


**Figure S9: Subgroup analyses for clinical reasoning**

**a)**

**
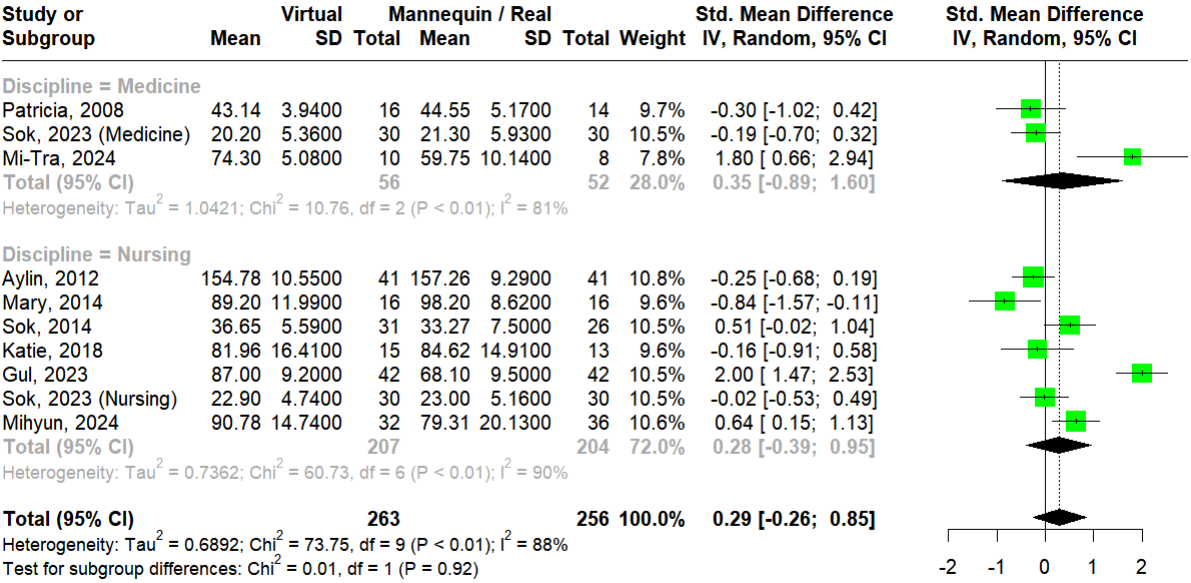
**

**b)**

**
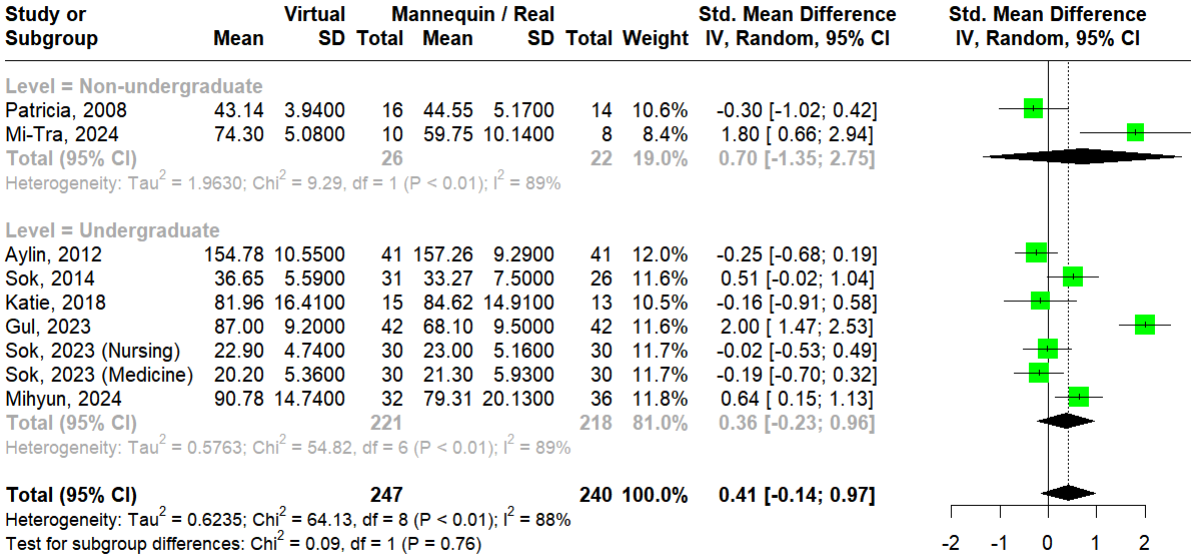
**

**c)**

**
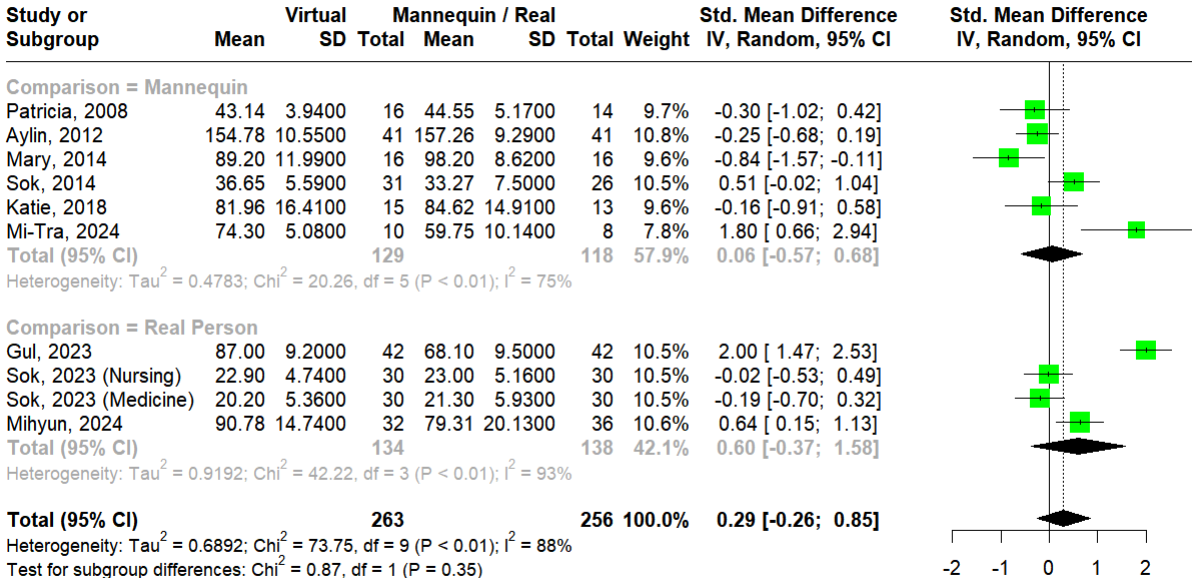
**

Subgroup analysis by a) discipline (medicine; nursing), b) level (undergraduate; non-undergraduate) and c) comparison (mannequin; real person).

**Figure S10: Trial sequential analysis for communication skills**


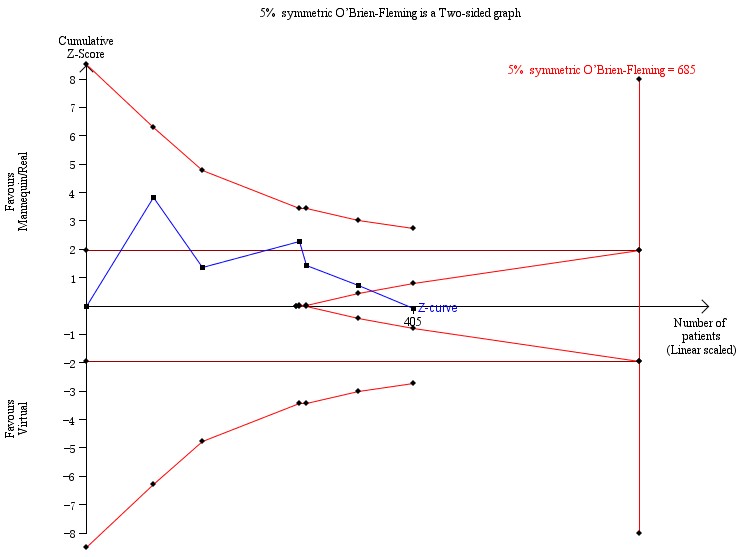


**Figure S11: Leave-one-out analysis for communication skills**


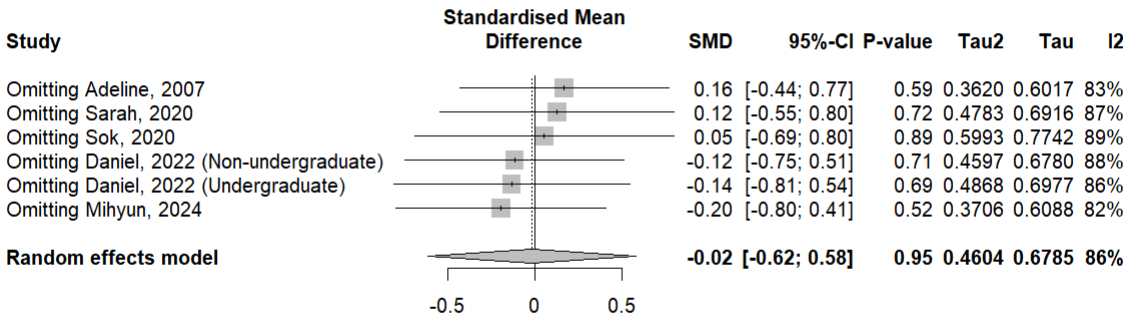


**Figure S12: Subgroup analysis for communication skills**

**a)**

**
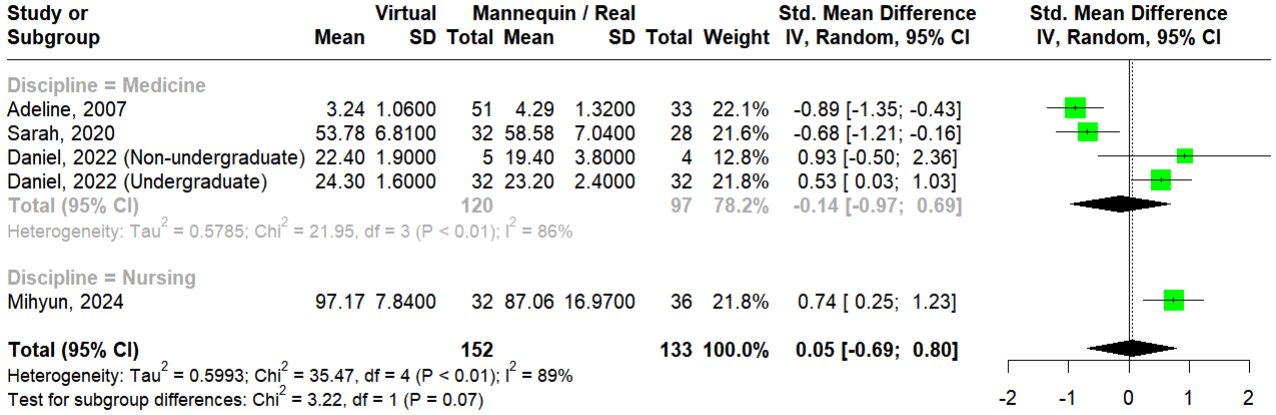
**

**b)**

**
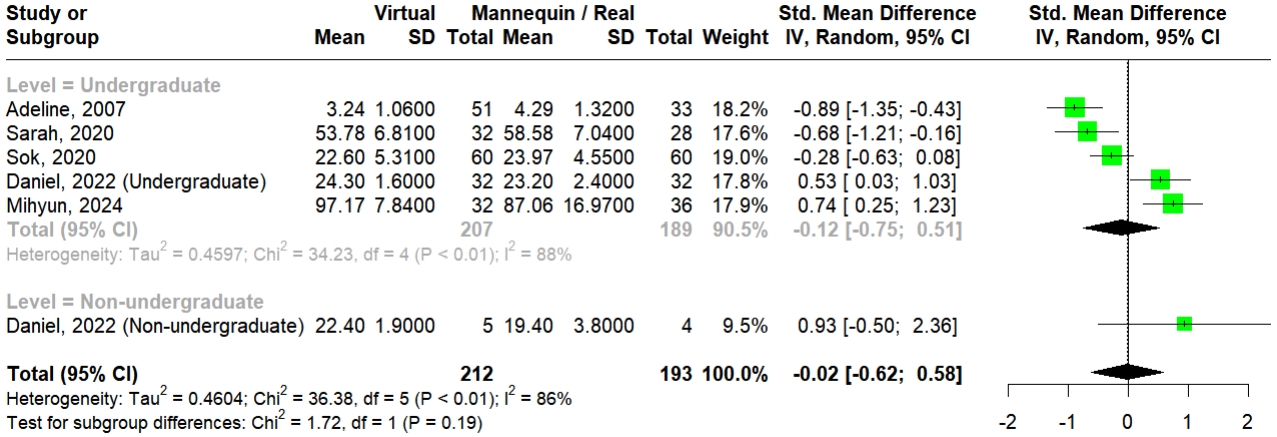
**

Subgroup analysis by a) discipline (medicine; nursing) and b) level (undergraduate; non-undergraduate).

**Table S1. Univariate random-effects meta-regression models by various covariates**

| **Outcome** | **Covariate** | **β** | **95% lower** | **95% upper** | **P-value** |
| --- | --- | --- | --- | --- | --- |
| **Knowledge** | Year of publication | 0.01 | -0.10 | 0.11 | 0.92 |
|  | Age of participants | -0.05 | -0.14 | 0.04 | 0.29 |
|  | Discipline: Nursing | 0.27 | -0.55 | 1.09 | 0.52 |
|  | Level: Undergraduate | 0.58 | -0.19 | 1.35 | 0.14 |
|  | Comparison: Real person | -0.25 | -1.15 | 0.64 | 0.58 |
| **Procedural skills** | Year of publication | -0.01 | -0.08 | 0.05 | 0.71 |
|  | Age of participants | 0.05 | -0.05 | 0.14 | 0.33 |
|  | Discipline: Nursing | -0.60 | -1.29 | 0.08 | 0.08 |
|  | Level: Undergraduate | -0.25 | -1.03 | 0.52 | 0.52 |
|  | Comparison: Real person | 0.19 | -0.54 | 0.92 | 0.61 |
| **Clinical reasoning** | Year of publication | 0.08 | -0.00 | 0.17 | 0.06 |
|  | Age of participants | -0.02 | -0.19 | 0.15 | 0.81 |
|  | Discipline: Nursing | -0.05 | -1.36 | 1.26 | 0.94 |
|  | Level: Undergraduate | -0.27 | -1.77 | 1.23 | 0.72 |
|  | Comparison: Real person | 0.53 | -0.59 | 1.65 | 0.35 |
| **Communication skills** | Year of publication | 0.09 | 0.01 | 0.16 | 0.02 |
|  | Age of participants | -0.07 | -0.75 | 0.61 | 0.84 |
|  | Discipline: Nursing | 0.88 | -0.89 | 2.66 | 0.33 |
|  | Level: Undergraduate | -1.05 | -3.10 | 1.00 | 0.32 |
|  | Comparison: Real person | — | — | — | — |
